# Bermudagrass mite (Acari: Eriophyidae) infestation worsens in response to increasing nitrogen fertility and decreasing irrigation volume but not mowing height

**DOI:** 10.1093/jee/toad205

**Published:** 2023-11-10

**Authors:** Matthew S Brown, Juang Horng Chong

**Affiliations:** Department of Entomology, Rutgers University, Thompson Hall, 96 Lipman Drive, New Brunswick, NJ 08901, USA; Department of Plant and Environmental Sciences, Clemson University, Pee Dee Research and Education Center, 2200 Pocket Road, Florence, SC 29506, USA

**Keywords:** mite, IPM, turf, cultural control

## Abstract

Severe bermudagrass mite (*Aceria cynodoniensis* Sayed) infestation stunts turfgrass growth and reduces the aesthetic and recreational value of managed bermudagrass. Management practices, such as fertilization, mowing, and irrigation, may impact bermudagrass mite infestation and damage, but empirical evidence is lacking. Two 20 wk experiments were conducted with potted bermudagrass in a greenhouse or nursery to evaluate the effect of varying nitrogen rates (0, 24.5, or 49 kg N/ha), mowing heights (1.3, 2.5, 3.8, or 5 cm), and irrigation rates (60%, 100%, or 140% evapotranspiration [ET] rate) on the densities of witch’s brooms (i.e., stunted and deformed terminals symptomatic of infestation) and bermudagrass mites. Increasing nitrogen fertility from 0 to 49 kg N/ha increased witch’s broom and bermudagrass mite densities by 292% and 339%, respectively. Bermudagrass fertilized with nitrogen maintained higher turf quality than unfertilized grass despite greater mite damage. Decreasing irrigation from 140% to 60% of the ET rate also increased witch’s broom densities by 124%. Mowing height did not consistently affect witch’s broom or mite densities. Witch’s broom and mite densities were positively correlated and followed a general trend with greater densities in April–August and a decline in densities in August–October. These findings suggest that nitrogen fertilization and water stress influence bermudagrass mite damage. Thus, limiting nitrogen fertilization to a level necessary to maintain turfgrass health and quality (0.5 kg N/ha) and minimizing turfgrass water stress can complement current chemical control strategies as part of an integrated pest management program.

Bermudagrass mites (*Aceria cynodoniensis* Sayed) are ~200 µm long and feed exclusively on bermudagrass (*Cynodon dactylon* [L.] Pers. [Poales: Poaceae] and *C. dactylon* × *C. transvaalensis* Burtt Davy) (Tuttle and Butler 1961). All bermudagrass mite lifestages live and develop under bermudagrass leaf sheaths and complete development from egg hatching to adult eclosion in 5–6 days (Butler 1963, Brown et al. 2021). Bermudagrass mite feeding induces abnormal growth and deformation, including shortened internodes, stunted leaves, and excessive tiller proliferation from a single node (Loch et al. 2017). Grass terminals displaying these abnormal growth symptoms are called witch’s brooms. Continuous bermudagrass mite infestation produces thinned and dead bermudagrass (Tuttle and Butler 1961). Turfgrass damaged by bermudagrass mites fails to meet the high aesthetic and playability standards required on golf courses and athletic fields. Bermudagrass mite infestation also impacts sod production when the weakened sod breaks during harvest, leading to production losses of up to 30% (Loch et al. 2017).

Turfgrass producers and managers attempt to manage bermudagrass mite infestations with pesticides, such as abamectin and pyrethroids (Brown et al. 2021). Additionally, they modify cultural practices, such as irrigation, fertilizer application, and aeration, to optimize turfgrass health and improve resilience to mite damage. However, current management practices are ineffective, and the management of severe infestations is rarely successful (Boeri et al. 2018). For example, there was only a small reduction in the number of witch’s brooms 36 days after abamectin application in turfgrass with a severe mite infestation, though abamectin reduced the number of witch’s brooms compared to a water-treated control (Boeri et al. 2018). Additionally, the most effective miticide (abamectin) is only available for applications to golf courses and professional and collegiate sports fields (Boeri et al. 2018). Therefore, turfgrass managers of other sports fields, sod farms, and residential landscapes must rely on cultural control strategies to manage bermudagrass mite.

Previous research and observations suggest that cultural or growing practices influence bermudagrass mite management (Tuttle and Butler 1961, Reinert et al. 2008); however, results have been inconsistent. Tuttle and Butler (1961) reported increased mite damage in lawns receiving fertilizers containing various nitrogen sources, including ammonium phosphate-sulfate, nitric phosphate, ammonium sulfate, fortified fish base liquid, urea-formaldehyde, and sewage sludge. In contrast, Reinert et al. (1978) reported that the number of witch’s brooms did not differ among bermudagrass sprigs grown in nutrient solution enriched with ammonium nitrate to nitrogen levels of 1.4, 14, 70, or 210 ppm. Fertilizers (ammonium sulfate and urea sulfur) combined with a miticide (diazinon) improved turfgrass quality and, sometimes, reduced damage compared to miticide alone (Butler et al. 1963, Butler and Stroehlein 1965).

Besides fertilization, evidence suggests mowing height and irrigation influence bermudagrass mite damage (Tuttle and Butler 1961, Johnson 1975, Reinert et al. 2008). Severe infestations are rarely observed on golf course greens, where turfgrass is mowed extremely short (0.25–0.64 cm; McCarty 2005) and frequently (Johnson 1975, Brown et al. 2021). Similarly, scalping turfgrass (i.e., mowing to remove aboveground plant tissues) may potentially control bermudagrass mite. However, combining scalping with a miticide application did not reduce witch’s broom density compared to a miticide alone (Boeri et al. 2018). In contrast, previous anecdotal observations indicate bermudagrass mite infestations are often found damaging turfgrass susceptible to water stress (i.e., slopes and nonirrigated turfgrass) and are less common on flood-irrigated lawns (Tuttle and Butler 1961, Reinert et al. 2008). These observations suggest that mowing height and water stress may influence the severity of mite infestation and damage.

In this study, greenhouse and nursery experiments were conducted to gather empirical data on whether manipulating nitrogen fertility rate, mowing height, and irrigation rate influences witch’s broom occurrence or mite densities. No study has examined the effect of fertilizer on bermudagrass mite density or at nitrogen rates typically applied on bermudagrass golf courses, where bermudagrass mite is a pest of great concern (Brown et al. 2021). We predicted that elevated nitrogen rates would increase mite density and damage due to increased nitrogen content in bermudagrass tissues, leading to improved mite fitness. No research has investigated the effect of mowing height or irrigation on bermudagrass mite infestation and subsequent damage. We predicted that maintaining a shorter bermudagrass height of cut would physically remove mites, reducing mite densities and damage. Based on previous observations indicating an association between water-stressed turfgrass and bermudagrass mite infestation, we predicted that reduced irrigation volume would increase mite density and damage.

## Materials and Methods

### Turfgrass Establishment and Mite Infestation

Greenhouse and nursery experiments were conducted at the Clemson University Pee Dee Research and Education Center (PDREC) in Florence, SC. Plugs (10.8 cm in diameter) of bermudagrass (*C*. *dactylon*, cv. ‘Celebration’) were collected from the Clemson University turfgrass research and training field in Clemson, SC, in May 2019. The soil was washed from the roots, and the soilless plugs were transplanted individually into 18-liter pots filled with sand/peat moss (85/15% v/v; USGA 2004). Potted bermudagrass was maintained outdoors on landscape fabric at a nursery at PDREC. After transplant, a water-soluble fertilizer (20−20−20 N−P−K, 0.02% B, 0.05% Cu, 0.10% Fe, 0.05% Mn, 0.001% Mo, and 0.05% Zn; Ultrasol 20−20−20 Water-Soluble Fertilizer Multi-Purpose Plus; SQM North America, Atlanta, GA) was applied at 24.5 kg N/ha weekly for 4 wk to promote turfgrass establishment and monthly after establishment until October to maintain grass growth. Potted turfgrass was irrigated daily for the first 2 wk after transplant and as needed for the remainder of the experiments. Potted turfgrass was mowed with handheld grass shears (GSL35; Black and Decker, Towson, MD) 3 times a week to a height of 2.5 cm.

After 4 wk of initial turfgrass establishment, bermudagrass was infested with bermudagrass mites using methods similar to Johnson (1975). Witch’s brooms were collected from ‘Celebration’ bermudagrass at De Bordieu Colony Golf Course in Georgetown, SC. Witch’s brooms were brought to the laboratory and examined under the microscope to confirm the presence of mites. Four witch’s brooms were placed on top of the turfgrass in each pot. As the deformed grass terminals dried, the bermudagrass mites dispersed from the witch’s brooms and infested the potted bermudagrass. Bermudagrass mites were allowed to establish on the plants until green-up the following spring when experiments were initiated.

Each experiment was carried out in a randomized complete block design in 2020 and repeated in 2021. Two groups of potted bermudagrass (Groups A and B) for each experiment and year were selected in the spring, and experiments were initiated (Experiment 1: 6 May 2020 and 28 April 2021; Experiment 2: 14 April 2020 and 22 April 2021; Experiment 3: 1 June 2020 and 15 May 2021). Each group of potted bermudagrass included 6 replications for each treatment separated into blocks spaced ~1.5 m apart. Groups A and B in each experiment were separated by ~3 m. Group A was used to evaluate plant and soil parameters (turfgrass quality, nutrient content, biomass, and soil moisture) and mite damage (witch’s brooms density). Group B was destructively sampled monthly throughout the study to estimate bermudagrass mite density. All experiments were terminated 20 wk after initiating nitrogen fertility, mowing height, or irrigation rate treatments.

### Experiment 1: Effects of Nitrogen Fertility

Nitrogen was applied to potted bermudagrass at 0, 24.5, or 49 kg N/ha every 4 wk. The experimental fertilization rates and frequency were based on the current recommendation for bermudagrass turf on golf course fairways of 24.5−50 kg N/ha every 4−16 wk, with lower rates applied more frequently and higher rates less frequently (McCarty 2005). Thus, the 24.5 kg N/ha rate was considered the recommended nitrogen rate, and the 49 kg N/ha was considered an elevated nitrogen rate. Nitrogen rates typically applied to golf course fairways were selected because bermudagrass mite is primarily a pest on golf courses, most commonly in the fairway and rough areas. Ammonium nitrate (NH_4_NO_3_) was used as the nitrogen source because the sand-based potting media does not support the urease activity necessary for breaking down urea into ammonium (the nitrogen form usable by turfgrass) to be an adequate nitrogen source (Zantua et al. 1977). Weekly, a modified Hoagland’s solution (containing 31 mg/liter P, 235 mg/liter K, 200 mg/liter Ca, 145 mg/liter S, 48 mg/liter Mg, 2.5 mg/liter Fe, 0.5 mg/liter B, 0.5 mg/liter Mn, 0.1 mg/liter Ni, 0.05 mg/liter Zn, 0.02 mg/liter Cu, 0.01 mg/liter Mo, and 0.77 mg/liter Cl) was applied at 500 ml/pot as a minus N solution to all treatments (Hoagland and Arnon 1938). A minus N solution was used instead of a combination fertilizer to ensure that only the nitrogen input varied among treatments, whereas other nutrients were constant. Potted turfgrass was mowed to a height of 2.5 cm and irrigated as needed.

Witch’s broom density in each pot in Group A was recorded ~24 h before treatments began and biweekly for 20 wk after that. Turfgrass quality was evaluated according to the scale established by the National Turfgrass Evaluation Program, i.e., on a scale of 1–9 with 1 = poor quality, 6 = minimum acceptable quality, and 9 = best quality based on visual assessments of color, density, uniformity, texture, and apparent symptoms of disease or environmental stress (Morris and Shearman 2012). The same person evaluated the visual quality throughout the experimental period to avoid deviation or differences among observers.

Plant biomass was determined at the end of the experiment. Plant shoots were harvested from Group A, and the roots were removed and washed. Plant tissues were dried in a drying oven at 50 °C for 7 days, and the dry shoot weight was recorded. The dried roots were weighed, placed in a furnace at 525 °C for 3 h, and the ash was weighed. The difference between dry root weight and ash weight is reported for root weights (Baldwin et al. 2009). Grass clippings were collected after mowing 20 wk after treatment initiation. The samples were sent to Clemson University Agricultural Service Laboratory (Clemson, SC) to determine the % nitrogen content in plant tissues.

From Group B, 3 witch’s brooms per pot were collected ~24 h before and every 4 wk after treatments began to determine mite density. The witch’s brooms were brought to the laboratory, and each sample (i.e., all witch’s brooms from each pot) was weighed before mite extraction with methods adapted from Monfreda et al. (2007) to determine mite densities (mites/mg of plant sample). Each sample was placed in a solution with 0.05% dish soap and 1% bleach (v/v), and the grass was shredded for ~10 s using an immersion blender. Then, the solution was poured through 2 stacked sieves: the top sieve (mesh size: 350 µm) to remove plant parts and the bottom sieve (mesh size: 20 µm) to collect mite eggs and motiles (nymphs and adults). Eggs and motiles were counted under stereomicroscopes (20× magnification). Mite nymphs and adults were combined into a single term, motiles because the 2 life stages cannot be differentiated without slide mounting.

Environmental data (air temperature, rainfall, and relative humidity) were obtained from a weather station ~2 km from the nursery. The average daily air temperature during the experimental periods ranged from 14.8 to 29.8 °C in 2020 and 16.2 to 33.2 °C in 2021. The average daily relative humidity ranged from 70 to 99% in 2020 and 39 to 97% in 2021. There were 62 rain events averaging 1.3 cm of precipitation/rain event in 2020 and 35 rain events averaging 1.4 cm of precipitation/rain event in 2021.

### Experiment 2: Effects of Mowing Height

Potted bermudagrass was cut to typical mowing heights on golf course fairways and roughs (1.3, 2.5, 3.8, or 5.0 cm). Bermudagrass fairways are usually maintained at mowing heights of 1.1–2.2 cm, and bermudagrass roughs at 1.9–7.6 cm (McCarty 2005). Potted bermudagrass was mowed with handheld grass shears. Dowel rods were cut the length of our mowing height treatments to serve as guides while mowing. A water-soluble 20−20−20 N−P−K fertilizer was applied at 24.5 kg N/ha every 4 wk, and potted turfgrass was irrigated as needed.

Witch’s broom density and turf quality were evaluated (as previously described) in each pot in Group A ~24 h before treatments began and biweekly for 20 wk after that. From Group B, 3 witch’s brooms were collected ~24 h before and every 4 wk after treatments began. The witch’s brooms were brought to the laboratory and processed to determine mite density as previously described.

Environmental data (air temperature, rainfall, and relative humidity) were obtained from a weather station ~2 km from the nursery. The average daily air temperature during the experimental periods ranged from 13.5 to 29.6 °C in 2020 and 13.1 to 33.2 °C in 2021. The average daily relative humidity ranged from 54% to 99% in 2020 and 39% to 97% in 2021. There were 62 rain events averaging 1.3 cm of precipitation/rain event in 2020 and 35 rain events averaging 1.4 cm of precipitation/rain event in 2021.

### Experiment 3: Effects of Irrigation Rate

Potted bermudagrass was irrigated with 60%, 100%, or 140% of the evapotranspiration (ET) rate. An irrigation treatment with 100% of the ET rate was used to represent optimal irrigation. Fu et al. (2004) reported that a 60% ET rate was the minimum water application requirement for acceptable turfgrass quality in bermudagrass. Thus, a 60% ET rate was set as a reduced irrigation rate treatment and a 140% ET rate as an elevated irrigation rate treatment.

Pots were transferred from the nursery to a greenhouse 2 wk before the experiment to maintain control of the prescribed ET rates. The ET rate was determined using the gravimetric mass balance method (Bowman and Macaulay 1991). All potted bermudagrass was saturated by thorough irrigation. To determine the weight of pots of bermudagrass at field capacity, potted bermudagrass assigned to the 100% ET rate treatment was weighed after drainage had stopped at ~1 h. At the next watering, the pots of bermudagrass assigned to the 100% ET rate treatment were re-weighed. The difference between the weight at re-weighing and the weight at field capacity was calculated. The water lost due to evapotranspiration caused the weight difference, which was converted to the volume of water lost because the density of water is approximately 1 g/ml, so 1 g water = 1 ml water. Then, the appropriate water volume (100% ET rate) was applied to pots assigned to the 100% ET rate to return them to the original field capacity. The described process was repeated, and the pots of bermudagrass assigned to the 100% ET rate treatment were re-weighed at every watering. From the water volume lost at each watering in the pots assigned to the 100% ET rate treatment, the irrigation volumes required for the other treatments (60% and 140% ET rate) were calculated. Then, the appropriate irrigation volume was applied to the potted bermudagrass assigned to those treatments. Plants were watered 3−4 times weekly to achieve 60, 100, or 140% of the calculated ET rate. To calculate the ET rate, the water volume lost was averaged over the days since the previous watering, and the water volume lost was divided by the turfgrass area to produce the water depth lost per day (mm/d). The daily ET rate ranged from 1.46 to 13.15 mm/day in 2020 and 4.12 to 14.64 mm/day in 2021. A water-soluble 20−20−20 N−P−K fertilizer was applied at 24.5 kg N/ha every 4 wk, and potted turfgrass was mowed to a height of 2.5 cm.

Witch’s broom density and turf quality were evaluated (as previously described) in each pot in Group A ~24 h before treatments began and biweekly for 20 wk after that. Plant biomass was determined at the end of the experiment as previously described. The volumetric soil water content (VSWC) (m^3^ of water/m^3^ of total soil volume) was recorded at 3.8 cm deep weekly with a soil moisture meter (TDR 150 Soil Moisture Meter, Spectrum Technologies, Aurora, IL) at 4 locations ~7 cm from the edge in each pot of bermudagrass. From Group B, 3 witch’s brooms were collected ~24 h before and every 4 wk after treatments began. The witch’s brooms were brought to the laboratory and processed to determine mite density as previously described.

In the greenhouse, we obtained environmental data (air temperature and relative humidity) from a data logger (HOBO logger MX Temp/RH logger MX1101, Onset, Bourne, MA). The average daily air temperature during the experimental periods ranged from 19.8 to 35.6 ℃ in 2020 and 24 to 35.1 ℃ in 2021. The average daily relative humidity ranged from 45 to 93% in 2020 and 39 to 97% in 2021.

### Data Analysis

A generalized linear mixed model (GLMM) with a negative binomial distribution and a log link function was used to test for an effect of the experimental treatments on witch’s broom density (numbers/pot) using R (version 4.2.2; R Core Team 2020) with the glmmTMB function in the “glmmTMB” package. Treatment, week, and block were assigned as fixed effects for each experiment, and the pot of turfgrass was set as a random intercept to account for the dependency from repeated-measures on the same pot of turfgrass at each observation date. The combined densities of eggs and motiles (numbers/mg of plant sample) data were analyzed using a GLMM similar to the analysis for the witch’s broom density, except using sample weight as an offset. The eggs and motiles were combined because preliminary graphs revealed similar patterns of treatment effects on egg and motile densities. Using data from all experiments, the association between the average witch’s broom densities and the average mite densities at each sampling week was assessed with Pearson’s correlation analysis.

The turfgrass quality rating data were tested for an effect of our experimental treatments within each sampling week using the nonparametric Friedman test with the friedman.test function. In Experiment 3, the VSWC data were analyzed for treatment effect with repeated-measures analysis of variance (ANOVA) using the anova_test function from the “rstatix” package. The % nitrogen content data in Experiment 1 and root and shoot biomass data in Experiments 1 and 3 were analyzed for treatment effect using ANOVA. For all analyses, α = 0.05 was set, and Tukey’s HSD was used to separate means if significant differences among treatments were detected. In analyses using a GLMM or repeated-measures ANOVA, treatments were compared within each week when a significant interaction between treatment and week was detected. Orthogonal polynomial contrasts were used to test for linear and quadratic effects of experimental treatments on nitrogen content and plant biomass at *P* ≤ 0.05.

## Results

### Experiment 1: Effects of Nitrogen Fertility

The nitrogen content (%) in grass clippings increased with increasing nitrogen fertility rates in 2020 (*F* = 17.36; df = 2, 15; *P* = 0.0018) and 2021 (*F* = 14.91; df = 2, 15; *P* < 0.0002). In 2020, the nitrogen content in bermudagrass tissues was 0.95 ± 0.05, 2.04 ± 0.24, and 3.13 ± 0.35 in the 0, 24.5, and 49 kg N/ha treatments, respectively, with a significant linear response (polynomial contrasts, *P* < 0.001). In 2021, the mean ± SE % nitrogen content in bermudagrass tissues was 1.27 ± 0.15, 2.42 ± 0.25, and 3.72 ± 0.34 in the 0, 24.5, and 49 kg N/ha treatments, respectively, with a significant linear response (polynomial contrasts, *P* < 0.0001).

In both years, there were significant differences in root (2020: *F* = 12.92; df = 2, 15; *P* = 0.0017; 2021: *F* = 17.98; df = 2, 15; *P* = 0.0005) and shoot (2020: *F* = 9.82; df = 2, 15; *P* = 0.0044; 2021: *F* = 8.15; df = 2, 15; *P* = 0.0080) biomass among nitrogen rate treatments. In 2020, the mean ± SE root biomass (g) was 194.2 ± 12.6, 247.9 ± 6.5, and 220.9 ± 8.8 in the 0, 24.5, and 49 kg N/ha treatments, respectively, with a significant linear response (polynomial contrasts, *P* < 0.01). In 2021, the mean ± SE root biomass (g) was 187.8 ± 13.5, 248.8 ± 7.2, and 203.9 ± 8.9 in the 0, 24.5, and 49 kg N/ha treatments, respectively, with a significant linear response (polynomial contrasts, *P* < 0.001). In 2020 the mean ± SE shoot biomass (g) was 71.4 ± 8.9, 96.9 ± 7.2, and 108.9 ± 6.3 in the 0, 24.5, and 49 kg N/ha treatments, respectively, with a significant linear response (polynomial contrasts, *P* < 0.01). In 2021, the mean ± SE shoot biomass (g) was 81.3 ± 8.3, 111.7 ± 7.2, and 114.8 ± 5.4 in the 0, 24.5, and 49 kg N/ha treatments, respectively, with a significant linear response (polynomial contrasts, *P* < 0.01). In both years, bermudagrass turf receiving the recommended nitrogen rate (24.5 kg N/ha) produced greater root biomass than unfertilized turfgrass (0 kg N/ha) and turfgrass receiving the elevated nitrogen rate (49 kg N/ha). In both years, turfgrass receiving the elevated nitrogen rate produced greater root and shoot biomass than the unfertilized turfgrass.

There were significant differences in turfgrass quality among nitrogen rate treatments on all sampling weeks in 2020 (Week 2: χ^2^ = 6.30; df = 2, 15; *P* = 0.0429; Week 4: χ^2^ = 8.86; df = 2, 15; *P* = 0.0119; Week 6: χ^2^ = 11.00; df = 2, 15; *P* = 0.0041; Week 8: χ^2^ = 8.44; df = 2, 15; *P* = 0.0147; Week 10: χ^2^ = 10.38; df = 2, 15; *P* = 0.0056; Week 12: χ^2^ = 10.38; df = 2, 15; *P* = 0.0056; Week 14: χ^2^ = 6.33; df = 2, 15; *P* = 0.0421; Week 16: χ^2^ = 11.27; df = 2, 15; *P* = 0.0036; Week 18: χ^2^ = 11.27; df = 2, 15; *P* = 0.0036; Week 20: χ^2^ = 10.57; df = 2, 15; *P* = 0.0051). In 2021, there were significant differences in turfgrass quality among nitrogen rate treatments on 4 out of 10 sampling weeks (Week 2: χ^2^ = 2.00; df = 2, 15; *P* = 0.3679; Week 4: χ^2^ = 3.44; df = 2, 15; *P* = 0.1787; Week 6: χ^2^ = 1.65; df = 2, 15; *P* = 0.4378; Week 8: χ^2^ = 0.82; df = 2, 15; *P* = 0.6643; Week 10: χ^2^ = 6.87; df = 2, 15; *P* = 0.0322; Week 12: χ^2^ = 7.91; df = 2, 15; *P* = 0.0191; Week 14: χ^2^ = 4.53; df = 2, 15; *P* = 0.1040; Week 16: χ^2^ = 6.33; df = 2, 15; *P* = 0.0421; Week 18: χ^2^ = 8.45; df = 2, 15; *P* = 0.0146; Week 20: χ^2^ = 5.73; df = 2, 15; *P* = 0.0571). The unfertilized treatment resulted in lower ratings than the recommended and elevated nitrogen rate on 6 out of 10 sampling weeks in 2020 and 1 sampling week in 2021. Unfertilized turfgrass maintained acceptable turf quality (rating ≥ 6), except on 2 out of 10 sampling weeks in 2020 and 3 out of 10 sampling weeks in 2021, whereas fertilized turfgrass maintained acceptable turfgrass quality throughout the experimental periods in both years.

**Fig. 1.**
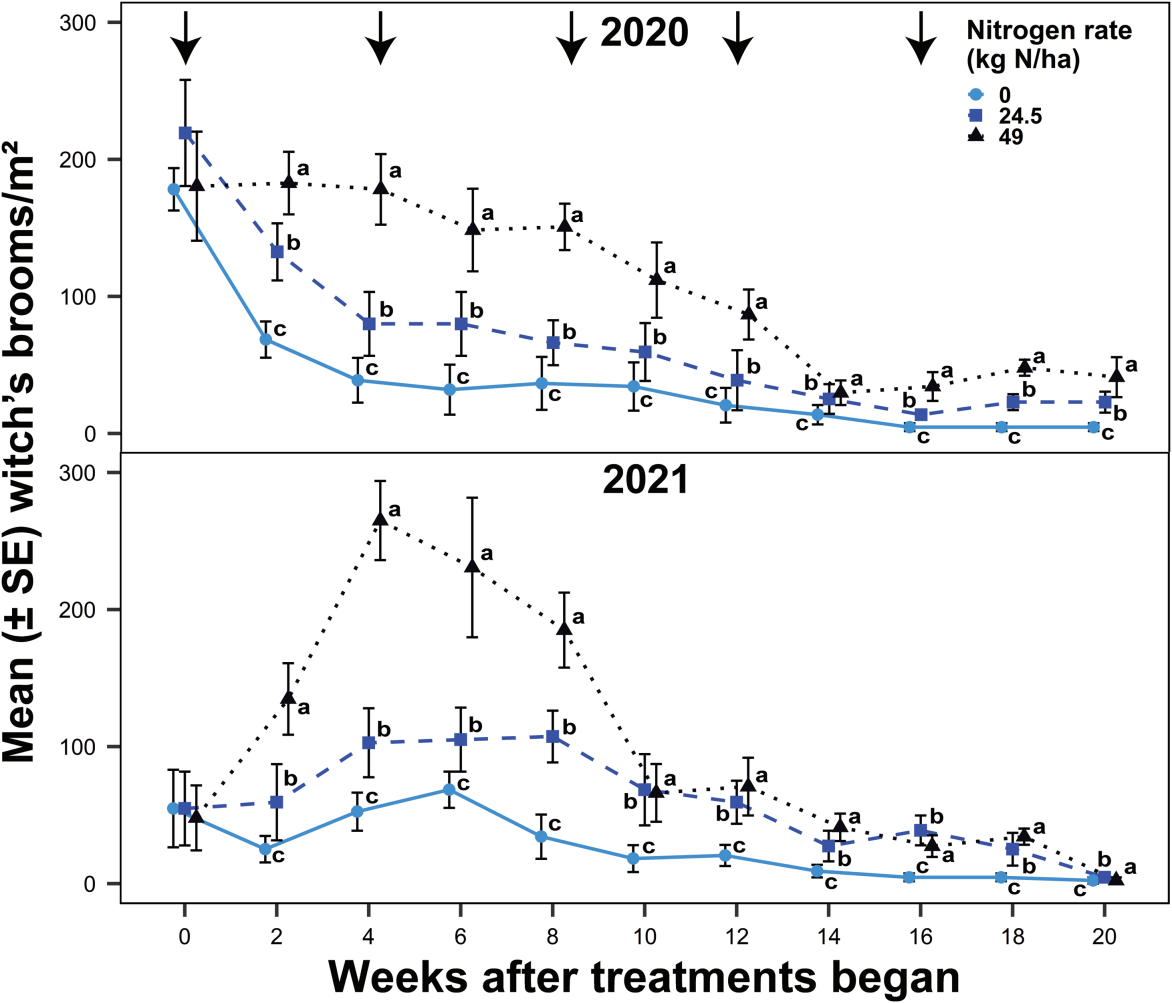
Witch’s broom densities (number/m^2^) in bermudagrass turfgrass fertilized at 0, 24.5, or 49 kg N/ha every 4 wk over 20 wk in 2020 and 2021. Arrows indicate the timing of nitrogen applications in fertilized treatments. Dots and bars (means ± SEM) at the same sampling week accompanied by different letters were significantly different among the nitrogen fertility treatments. Tukey’s HSD was used to separate means at α = 0.05.

There were significant differences in witch’s broom densities among nitrogen rate treatments in 2020 (χ^2^ = 11.39; df = 2, 143; *P* = 0.0034) and 2021 (χ^2^ = 19.43; df = 2, 143; *P* < 0.0001). Also, there were significant differences in witch’s broom densities among sampling weeks (2020: χ^2^ = 42.97; df = 9, 143; *P* < 0.0001; 2021: χ^2^ = 45.86; df = 9, 143; *P* < 0.0001), with no significant interaction between nitrogen rate and sampling week (2020: χ^2^ = 11.87; df = 18, 143; *P* = 0.8539; 2021: χ^2^ = 17.56; df = 18, 143; *P* = 0.4850). In 2020, witch’s broom densities increased with increasing nitrogen fertility rate (Fig. 1). The elevated nitrogen rate treatment increased witch’s broom densities by 87 and 293% compared to the recommended and unfertilized treatments, respectively. The recommended nitrogen rate treatment increased witch’s broom densities by 110% compared to the unfertilized treatment. In 2021, fertilization (both rates) increased witch’s broom densities compared to the unfertilized treatments, and there was no difference between the fertilized treatments (Fig. 1). The elevated and recommended nitrogen rate treatments increased witch’s broom densities by 341 and 150%, respectively, compared to the unfertilized treatment.

**Fig. 2.**
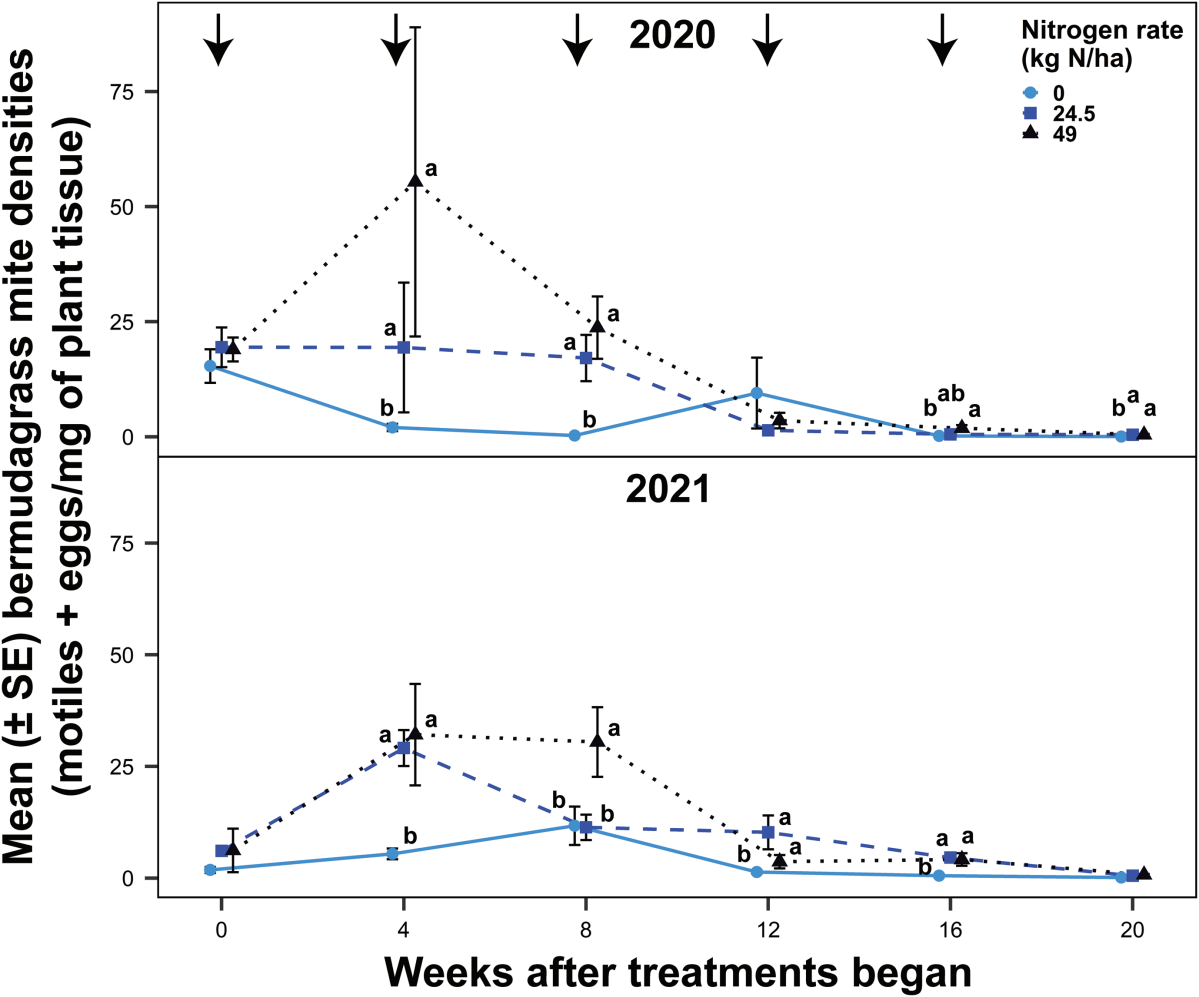
Bermudagrass mite densities (motiles + eggs/mg of plant tissue) in bermudagrass turfgrass fertilized at 0, 24.5, or 49 kg N/ha every 4 wk over 20 wk in 2020 and 2021. Arrows indicate the timing of nitrogen applications in fertilized treatments. Three witch’s brooms were collected from each pot on each sampling week. Plant samples were weighed, and the numbers of motiles and eggs were counted using a microscope. Dots and bars (means ± SEM) at the same sampling week accompanied by different letters were significantly different among the nitrogen fertility treatments. Tukey’s HSD was used to separate means at α = 0.05.

In both years, there were significant differences in mite densities among nitrogen rate treatments (2020: χ^2^ = 21.65; df = 2, 68; *P* < 0.0001; 2021: χ^2^ = 26.37; df = 2, 68; *P* < 0.0001) and sampling weeks (2020: χ^2^ = 52.71; df = 4, 68; *P* < 0.0001; 2021: χ^2^ = 140.23; df = 4, 68; *P* < 0.0001) and a significant interaction between nitrogen rate and sampling week (2020: χ^2^ = 47.93; df = 8, 68; *P* < 0.0001; 2021: χ^2^ = 25.77; df = 8, 68; *P* < 0.0001). The unfertilized treatment resulted in lower mite densities than fertilization (both rates) in 3 out of 5 sampling weeks in 2020 and 4 out of 5 sampling weeks in 2021 (Fig. 2). In 2020, the elevated and recommended nitrogen rate treatments resulted in a 646 and 254% increase in mite densities, respectively, compared to the unfertilized treatment. In 2021, the elevated and recommended nitrogen rate treatments resulted in a 282 and 247% increase in mite densities, respectively, compared to the unfertilized treatment.

### Experiment 2: Effects of Mowing Height

There were no significant differences in turfgrass quality among mowing height treatments on any sampling week in 2020 (Week 2: χ^2^ = 3.16; df = 3, 20; *P* = 0.3671; Week 4: χ^2^ = 1.85; df = 3, 20; *P* = 0.6031; Week 6: χ^2^ = 0.43; df = 3, 20; *P* = 0.9343; Week 8: χ^2^ = 2.45; df = 3, 20; *P* = 0.4836; Week 10: χ^2^ = 1.29; df = 3, 20; *P* = 0.7325; Week 12: χ^2^ = 2.00; df = 3, 20; *P* = 0.5724; Week 14: χ^2^ = 4.33; df = 3, 20; *P* = 0.2276; Week 16: χ^2^ = 0.66; df = 3, 20; *P* = 0.8826; Week 18: χ^2^ = 0.06; df = 3, 20; *P* = 0.9959; Week 20: χ^2^ = 2.06; df = 3, 20; *P* = 0.5603). In 2021, there were significant differences in turfgrass quality among mowing height treatments on 3 out of 10 sampling weeks (Week 2: χ^2^ = 5.22; df = 3, 20; *P* = 0.1564; Week 4: χ^2^ = 8.06; df = 3, 20; *P* = 0.0451; Week 6: χ^2^ = 7.14; df = 3, 20; *P* = 0.0676; Week 8: χ^2^ = 0.89; df = 3, 20; *P* = 0.8287; Week 10: χ^2^ = 2.61; df = 3, 20; *P* = 0.4555; Week 12: χ^2^ = 1.79; df = 3, 20; *P* = 0.6174; Week 14: χ^2^ = 11.14; df = 3, 20; *P* = 0.0110; Week 16: χ^2^ = 12.05; df = 3, 20; *P* = 0.0072; Week 18: χ^2^ = 6.41; df = 3, 20; *P* = 0.0932; Week 20: χ^2^ = 3.88; df = 3, 20; *P* = 0.2745). In 2021, bermudagrass turf mowed to 1.3 cm was rated at a higher quality than turfgrass mowed to 2.5 cm at Weeks 4, 14, and 16.

**Fig. 3.**
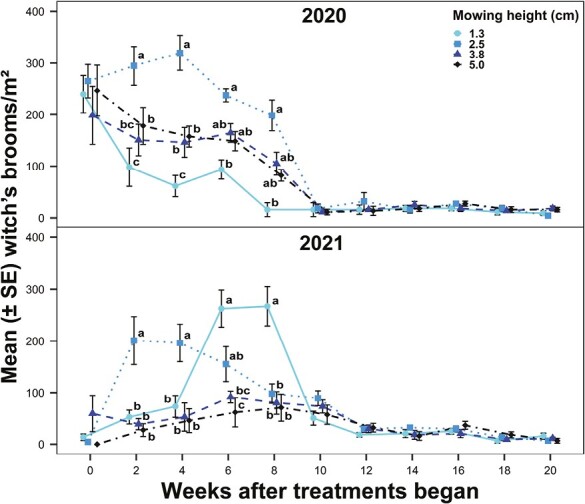
Witch’s broom densities (number/m^2^) in bermudagrass turf maintained at 1.3, 2.5, 3.8, or 5.0 cm over 20 wk in 2020 and 2021. Dots and bars (means ± SEM) at the same sampling week accompanied by different letters were significantly different among the mowing height treatments. Tukey’s HSD was used to separate means at α = 0.05.

The response of witch’s broom densities to mowing height was inconsistent over the 2 yr. There were significant differences in witch’s broom densities among mowing height treatments (2020: χ^2^ = 31.68; df = 3, 193; *P* < 0.0001; 2021: χ^2^ = 48.85; df = 3, 193; *P* < 0.0001) and sampling weeks (2020: χ^2^ = 101.09; df = 9, 193; *P* < 0.0001; 2021: χ^2^ = 176.19; df = 9, 193; *P* < 0.0001) and a significant interaction between mowing height and sampling week in both years (2020: χ^2^ = 47.69; df = 27, 193; *P* = 0.0083; 2021: χ^2^ = 108.87; df = 27, 193; *P* < 0.0001). In 2020, mowing to 2.5 cm resulted in greater witch’s broom densities than mowing to the 2 highest heights of cut at Weeks 2 and 4 (April−May) and greater witch’s broom densities than 1.3 cm at Weeks 2–8 (April−June) (Fig. 3). Similarly, in 2021, mowing to 2.5 cm resulted in greater witch’s broom densities than other mowing heights at Weeks 2 and 4 (May). Mowing to 1.3 cm resulted in greater witch’s broom densities than mowing to the 2 highest heights of cut at Weeks 6 and 8 (June), which differed from 2020, when mowing to 1.3 cm often resulted in lower witch’s broom densities.

For both years, mite densities were not significantly different among mowing height treatments (2020: χ^2^ = 0.90; df = 3, 93; *P* = 0.8245; 2021: χ^2^ = 3.57; df = 3, 93; *P* = 0.3115) but were significantly different among sampling weeks (2020: χ^2^ = 62.22; df = 4, 93; *P* < 0.0001; 2021: χ^2^ = 54.74; df = 4, 93; *P* < 0.0001). There was no significant interaction between mowing height and sampling week (2020: χ^2^ = 18.19; df = 12, 93; *P* = 0.1100; 2021: χ^2^ = 15.18; df = 12, 93; *P* = 0.2319). In 2020, the mean ± SE mite density (motiles + eggs/mg of plant tissue) was 184 ± 53, 289 ± 67, 358 ± 67, and 204 ± 69 in the 1.3, 2.5, 3.8, and 5.0 cm mowing height treatments, respectively. In 2021, the mean ± SE mite density (motiles + eggs/mg of plant tissue) was 231 ± 108, 262 ± 140, 486 ± 305, and 338 ± 197 in the 1.3, 2.5, 3.8, and 5.0 cm mowing height treatments, respectively.

### Experiment 3: Effects of Irrigation Rate

In both years, there were significant differences in VSWC among irrigation rate treatments (2020: *F* = 411.90; df = 2, 1372; *P* < 0.0001; 2021: *F* = 1547.98; df = 2, 1372; *P* < 0.0001) and sampling weeks (2020: *F* = 49.53; df = 19, 1372; *P* < 0.0001; 2021: *F* = 127.74; df = 19, 1372; *P* < 0.0001) and a significant interaction between irrigation rate and sampling week (2020: *F* = 5.20; df = 38, 1372; *P* < 0.0001; 2021: 6.20; df = 38, 1372; *P* < 0.0001). The reduced irrigation rate treatment (60% ET rate) lowered the VSWC compared to the recommended (100% ET rate) and the elevated irrigation rate (140% ET rate) after 4 wk in 2020 and after 1 wk in 2021. The recommended irrigation rate treatment resulted in lower VSWC than the elevated irrigation rate on 7 out of 20 sampling weeks in 2020 and 9 out of 20 sampling weeks in 2021.

Root biomass was significantly different among irrigation rate treatments in 2020 (*F* = 6.82; df = 2, 15; *P* = 0.0135) and 2021 (*F* = 4.27; df = 2, 15; *P* = 0.0458), and shoot biomass was significantly different in 2021 (*F* = 4.28; df = 2, 15; *P* = 0.0454) but not 2020 (*F* = 0.69; df = 2, 15; *P* = 0.5253). In 2020, the mean ± SE root biomass (g) was 100.7 ± 4.8, 141.6 ± 10.8, and 107.4 ± 12.1 in the 60, 100, and 140% ET rate treatments, respectively, with a significant linear response (polynomial contrasts, *P* < 0.01). In 2021, the mean ± SE root biomass (g) was 110.2 ± 5.5, 150.8 ± 10.8, and 122.6 ± 13.3 in the 60, 100, and 140% ET rate treatments, respectively, with a significant linear response (polynomial contrasts, *P* < 0.05). In 2020, the mean ± SE shoot biomass (g) was 84.5 ± 9, 91.1 ± 6.3, and 94.5 ± 9.6 in the 60, 100, and 140% ET rate treatments, respectively, with no significant linear or quadratic response. In 2021, the mean ± SE shoot biomass (g) was 85.2 ± 5.3, 106.4 ± 6.1, and 104.5 ± 8.3 in the 60, 100, and 140% ET rate treatments, respectively, with no significant linear or quadratic response. Bermudagrass receiving the reduced irrigation rate produced lower root biomass than turfgrass receiving the recommended irrigation rate in both year s and shoot biomass in 2021.

There were significant differences in turfgrass quality among irrigation rates on 7 out of 10 sampling weeks in 2020 (Week 2: χ^2^ = 3.00; df = 2, 15; *P* = 0.2231; Week 4: χ^2^ = 9.58; df = 2, 15; *P* = 0.0083; Week 6: χ^2^ = 3.50; df = 2, 15; *P* = 0.1738; Week 8: χ^2^ = 11.27; df = 2, 15; *P* = 0.0036; Week 10: χ^2^ = 4.53; df = 2, 15; *P* = 0.1040; Week 12: χ^2^ = 7.36; df = 2, 15; *P* = 0.0252; Week 14: χ^2^ = 11.57; df = 2, 15; *P* = 0.0031; Week 16: χ^2^ = 11.00; df = 2, 15; *P* = 0.0041; Week 18: χ^2^ = 11.57; df = 2, 15; *P* = 0.0031; Week 20: χ^2^ = 6.12; df = 2, 15; *P* = 0.0469). The reduced irrigation treatment led to lower ratings than the recommended and elevated irrigation rates on 4 of 10 sampling weeks. In 2021, there were significant differences in turfgrass quality among irrigation rates on 4 out of 10 sampling weeks (Week 2: χ^2^ = 0.3529; df = 2, 15; *P* = 0.8382; Week 4: χ^2^ = 4.57; df = 2, 15; *P* = 0.1017; Week 6: χ^2^ = 0.10; df = 2, 15; *P* = 0.9535; Week 8: χ^2^ = 10.33; df = 2, 15; *P* = 0.0057; Week 10: χ^2^ = 8.28; df = 2, 15; *P* = 0.0160; Week 12: χ^2^ = 4.96; df = 2, 15; *P* = 0.0839; Week 14: χ^2^ = 2.70; df = 2, 15; *P* = 0.2598; Week 16: χ^2^ = 9.48; df = 2, 15; *P* = 0.0087; Week 18: χ^2^ = 4.36; df = 2, 15; *P* = 0.1128; Week 20: χ^2^ = 9.00; df = 2, 15; *P* = 0.0111). The reduced irrigation treatment led to lower ratings than the recommended and elevated irrigation rates on 3 out of 10 sampling weeks. The quality of bermudagrass turf receiving the reduced irrigation rate was rated below minimally acceptable on 2 of 10 sampling weeks in 2020 and 5 out of 10 sampling weeks in 2021.

**Fig. 4.**
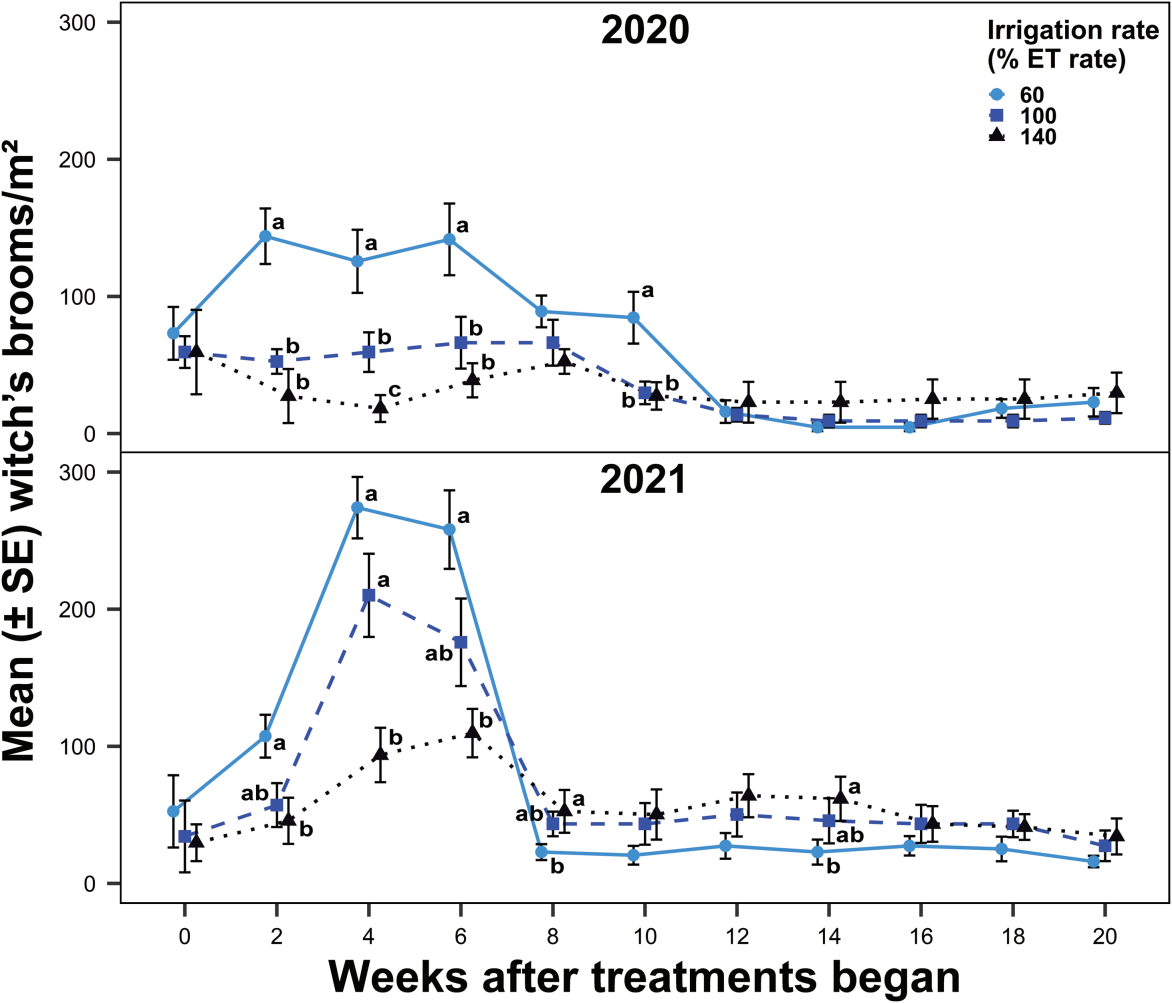
Witch’s broom densities (number/m^2^) in bermudagrass turf irrigated at 60%, 100%, or 140% evapotranspiration (ET) rate over 20 wk in 2020 and 2021. Dots and bars (means ± SEM) at the same sampling week accompanied by different letters were significantly different among the irrigation rate treatments. Tukey’s HSD was used to separate means at α = 0.05.

In both years, there were significant differences in witch’s broom densities among irrigation rate treatments (2020: χ^2^ = 27.19; df = 2, 143; *P* < 0.0001; 2021: χ^2^ = 7.30; df = 2, 143; *P* = 0.0260) and sampling weeks (2020: χ^2^ = 123.65; df = 9, 143; *P* < 0.0001; 2021: χ^2^ = 247.87; df = 9, 143; *P* < 0.0001) and a significant interaction between irrigation rate and sampling week (2020: χ^2^ = 66.19; df = 18, 143; *P* < 0.0001; 2021: χ^2^ = 57.35; df = 18, 143; *P* < 0.0001). In 2020, the reduced irrigation rate treatment resulted in greater witch’s broom densities than the recommended and elevated irrigation rates at Weeks 2, 4, 6, and 10 (June−August) (Fig. 4). In 2021, the reduced irrigation rate treatment increased witch’s broom densities compared to the elevated irrigation rate at Weeks 2−6 (May–June). Witch’s broom densities were not different between irrigation rates at Weeks 12–20 (August–October) in 2020 and Weeks 10–20 (July–October) (except Week 14) in 2021. In 2020, the reduced irrigation rate treatment resulted in 100 and 124% more witch’s brooms over the experimental period than the recommended and elevated irrigation rates, respectively. In 2021, the reduced irrigation rate treatment resulted in 8 and 34% greater witch’s broom densities over the experimental period than the recommended and elevated irrigation rates, respectively.

There were no significant differences in mite densities among irrigation rate treatments in either year (2020: χ^2^ = 5.14; df = 2, 68; *P* = 0.0765; 2021: χ^2^ = 3.86; df = 2, 68; *P* = 0.1454), but there were significant differences among sampling weeks and a significant interaction between irrigation rate and sampling week in 2021 (sampling week: χ^2^ = 60.41; df = 4, 68; *P* < 0.0001; interaction: χ^2^ = 26.70; df = 8, 68; *P* = 0.0008) but not 2020 (sampling week: χ^2^ = 4.17; df = 4, 68; *P* = 0.3838; interaction: χ^2^ = 4.54; df = 8, 68; *P* = 0.8052). In 2021, the elevated irrigation rate treatment reduced mite densities compared to the reduced irrigation rate on 1 sampling week and the recommended irrigation rate on 2 sampling weeks.

Using data from all experiments, the average witch’s broom densities and average mite densities at each sampling week were significantly positively associated (*r* = 0.78, df = 34, *P* < 0.0001).

## Discussion

In this study, increasing nitrogen fertilization rates and reducing irrigation rates worsened mite-induced damage in most sampling weeks, whereas mowing height did not produce a consistent effect. The effects of nitrogen fertilization, irrigation, and mowing were also influenced by the week and year the surveys were conducted, indicating that seasonal activity or environmental factors were a dominant influence on bermudagrass mite densities and damage. In most experiments, witch’s broom and mite densities followed a general trend with greater densities early in the first half of the experiments (Weeks 0–12; April–August) and a decline in densities in the second half of the experiments (Week 14–20; August–October). A similar trend in bermudagrass mite population dynamics has been observed on golf courses in North and South Carolina (Brown and Chong, unpublished data).

In the experiments conducted in the nursery (Experiments 1 and 2), environmental variables like temperature and wind speed may be responsible for the seasonal effects observed in our study. When mite densities and damage were greatest in Weeks 0–8 (April–July), the average ambient temperature was lower (2020: 20 and 23 °C; 2021: 23 and 24 °C) compared to Weeks 8–20 (July–October) (26 °C in 2020 and 2021), and the wind speed was higher in Weeks 0–8 (2020: 1.21 and 1.53 m/s; 2021: 1.05 and 1.14 m/s) than later in experiments (2020: 1.06 and 0.99 m/s; 2021: 1.01 and 1.00 m/s). These patterns suggest that temperature and wind speed may play a role in the seasonal activity of mites and mite damage observed in our study. However, other factors like daylength, plant phenology, or mite colonization patterns may be important. A longer period than the 2 yr our study was conducted is necessary to identify factors influencing the seasonal patterns of bermudagrass mites and their damage. Rainfall was 81.3 and 80.3 cm in 2020 and 50.3 and 49.5 cm in 2021, and average relative humidity was 83.8 and 81.9% in 2020 and 80.2 and 79.5% in 2021. Mite damage increased in the dryer year (2021) early in experiments but decreased in the wetter year (2020), indicating reduced rainfall and relative humidity may have led to increased mite damage and inconsistency in treatment effects between years. In Experiment 3, hotter temperatures and drier conditions in 2021 (temperature: 31 °C; relative humidity: 64%) than in 2020 (temperature: 30 °C; relative humidity: 68%) may have led to a steeper increase in mite damage early in experiments and inconsistency in treatment effects between years.

Bermudagrass turf increases new tiller growth after green-up in spring and summer. Increased new tiller growth may be responsible for the high levels of bermudagrass mite infestation observed in the spring and summer during this study by providing a higher density of terminals susceptible to mite infestation. Additionally, increased new tiller growth may provide optimal conditions for mite population growth, considering eriophyid mites, like bermudagrass mite, feed on meristematic tissue (Boczek and Shevchenko 1996), which is abundant during periods of new tiller growth. Further, increased new tiller growth may lead to increased expression of witch’s brooming symptoms. Bermudagrass mites are often observed on the meristematic tissue of auxiliary buds, even on asymptomatic shoots (Brown and Chong, unpublished data). These infested buds develop into new tillers, indicating that new tiller growth may be necessary for the development of witch’s brooms. Since witch’s brooms develop from new tillers, the abundance of new tiller growth in spring may increase the expression of symptoms.

Consistent with our expectations and Tuttle and Butler’s (1961) observations, increasing nitrogen fertilization rate increased witch’s broom densities. In contrast to our results, Reinert et al. (1978) showed that the numbers of plants with witch’s brooms did not differ among nitrogen rates. The discrepancy between the results of our study and those of Reinert et al. (1978) may be due to differences in the plant maintenance methods. Our experiments were conducted on potted bermudagrass, which allowed matting (i.e., lateral extension, crossing, and overlapping of stolons to form a mat of grass) of the turfgrass and the production of new tillers throughout the experiment. Reinert et al. (1978) conducted an experiment on individual sprigs kept in a nutrient solution, which did not allow lateral extension of stolons or the development of many new tillers. As previously mentioned, new tiller production may influence the production of witch’s brooms. Since nitrogen application increases the number of new tillers (Premati et al. 2003), the increased production of new tillers with nitrogen application may have led to greater witch’s broom densities in our study. Thus, elevated nitrogen application rates may trigger witch’s broom development by stimulating tiller production.

The effect of increasing nitrogen fertilization rate on bermudagrass mite density has yet to be explored previously. Consistent with our expectation, increasing nitrogen increased bermudagrass mite densities in our study, although the effect of nitrogen rate treatments was not consistent. Many herbivores have increased growth and reproduction when feeding on plant tissues with a higher nitrogen content (Mattson 1980). For example, the population growth rate of the twospotted spider mite (*Tetranychus urticae* Koch [Acari: Tetranychidae]) increased with increasing nitrogen application rate to strawberries (Alizade et al. 2016). The population growth rate of the wheat curl mite (*Aceria tosichella* Keifer) increased in response to nitrogen fertilization when mites contained wheat streak mosaic virus but not when mites were virus-free (Miller et al. 2015). In contrast, urea-based fertilization did not affect eriophyid mite population abundance on timothy grass (Dively et al. 2022). Our study indicates nitrogen fertilization increased the nitrogen content in bermudagrass tissues, potentially improving host quality and enhancing mite population growth. Larger bermudagrass mite populations might have also been responsible for the observed increases in the witch’s broom densities.

Based on our results, nitrogen application worsens mite infestations in bermudagrass turf. However, nitrogen application is necessary to maintain turfgrass health and quality, especially during green-up and the spring when witch’s broom and mite densities are greatest (Brown and Chong, unpublished data). Increased nitrogen improves turf quality by enhancing turfgrass density and color (Carrow et al. 1987). For example, the unfertilized treatment in our study resulted in turfgrass quality ratings consistently below fertilized turf and at unacceptable levels for 2–3 wk. These transient declines in turf quality would likely be important on high-value turf like golf course tees and fairways but not roughs or out-of-play turf where tolerance for mite damage is higher. Therefore, limiting the nitrogen rate at each application instead of withholding nitrogen will achieve a compromise between maintaining turfgrass health and limiting the development of mite damage. In our study, the recommended nitrogen fertilization rate (24.5 kg N/ha every 4 wk) maintained similar turfgrass quality as the elevated nitrogen rate (49 kg N/ha) while reducing witch’s broom densities by nearly half. A nitrogen fertilization rate that maintains turfgrass health and quality while avoiding excessive fertilization that exacerbates mite infestation should be selected for mite-infested turfgrass. Considering turfgrass quality was similar between nitrogen fertilization rates, a nitrogen rate above 24.5 kg N/ha every 4 wk is excessive. Nitrogen rates lower than 24.5 kg N/ha may further limit mite infestations, though more research is necessary to identify the minimum rate required to maintain turfgrass quality.

The relationship between mite damage (i.e., witch’s brooms), mite densities, and turfgrass quality is poorly understood. Witch’s broom densities and mite densities were positively associated in our study. Our results indicate that nitrogen fertilization improved turfgrass quality despite increased mite damage. Higher turfgrass quality alongside increased mite damage is surprising, considering mite-infested turfgrass on golf courses is often low quality with thinning and bare spots. Many factors likely affect the influence of mite densities and witch’s brooms on turfgrass quality. For example, mite infestation may only affect turf quality at certain times of the year, during green-up in the spring when mite infestation prevents stolons from growing laterally and matting. Or mite infestation may only reduce turfgrass quality over the long term (i.e., several years), which was not observed in our study that lasted only 5 mo.

Increasing fertilizer application in mite-infested areas may be desirable for improving turfgrass regrowth and recovery from damage or boosting plant tolerance to mite-induced damage. Indeed, increasing nitrogen rates improved turfgrass quality in our experiments. Nitrogen fertilization also increased root biomass, indicating the potential for enhanced regrowth from mite damage by improving water and nutrient absorption from the soil. If turfgrass managers desire this approach, they should delay fertilizer application until infestation levels have subsided or combine fertilizer with a pesticide application. Reducing bermudagrass mite densities may be necessary before or during fertilization to counter the positive effects of increased nitrogen fertilization on mite densities and damage identified in our study.

Mowing height had an inconsistent effect on witch’s broom densities and did not affect bermudagrass mite densities. We expected shorter mowing heights would physically remove bermudagrass mites and infested/damaged terminals, reducing witch’s broom densities. Scalping turfgrass has been suggested for bermudagrass mite control based on the hypothesis that it physically removes mites (Brown et al. 2021). However, shorter mowing heights did not reduce witch’s broom or mite densities. The 2.5-cm mowing height had the greatest witch’s broom densities in both years, while the 1.3-cm mowing height had the lowest densities in 2020 and the greatest densities in 2021. The increased witch’s broom densities may be related to the increased production of new tillers at shorter mowing heights (Sheffer et al. 1978, Lush and Rogers 1992). Shorter mowing height (below 2.5 cm) increases tiller production by reducing the plant hormone auxin to levels below that necessary to inhibit tiller production (McCarty 2005). Inconsistency between years may be due to differences in the development of mite infestation throughout the experiment. In 2020, witch’s broom densities declined over 20 wk, while in 2021, witch’s broom densities increased through the first 8 wk and then declined. Thus, mowing short (below 2.5 cm) may mainly increase susceptibility to mites during the escalation of mite damage throughout the season. Mite infestations are rare on golf course greens but possibly not due to short mowing height. Greens are managed more intensively than other areas of golf courses with increased irrigation, fertilization, and pesticide applications, which may contribute to lower stress, greater turfgrass growth, and scarcity of mite infestations on greens. Also, bermudagrass cultivars used on greens differ from those used on other golf course areas. The growth habit (i.e., shorter internodes and thinner stems) of ultradwarf cultivars used on greens may contribute to the inability of bermudagrass mites to colonize greens. However, these cultivars support severe mite infestations when mowed at higher heights of cut (Reinert et al. 2008).

In this study, increasing the irrigation rate decreased witch’s broom densities but did not affect mite densities. Consistent with our expectations, the reduced irrigation treatment resulted in greater witch’s broom densities than other treatments. Other mite species, including *Tetranychus evansi* Baker and Pritchard and *Aculops lycopersici* (Massee) (Acari: Eriophyidae), had increased population growth and caused more damage to water-stressed plants (Ximénez-Embún et al. 2016, 2017). Similarly, twospotted spider mites developed faster, dispersed from leaves less often, and laid more eggs on water-stressed plants (Migeon et al. 2023). Drought-susceptible corn hybrids supported greater Banks grass mite (*Oligonychus pratensis* [Banks] [Acari: Tetranychidae]) populations under water-stress irrigation compared to optimal irrigation (Ruckert et al. 2021). Drought conditions can benefit mite populations by raising canopy temperature, improving host nutritional quality (increased levels of free sugars and amino acids), suppressing plant defenses against mites, or a combination of these factors (Toole et al. 1984, Ximénez-Embún et al. 2017).

Irrigating at 60% of the ET rate often increased mite damage in bermudagrass turf. Therefore, using a greater than 60% ET rate replacement strategy in mite-infested areas prone to underwatering may prevent water stress-induced increases in mite damage. Additionally, monitoring for mite infestations should focus on areas most susceptible to water stress, such as out-of-play/use turfgrass, or with poor water retention, such as slopes or with sandy soil, and periods of low rainfall/high evapotranspiration demand. Replacing bermudagrass in out-of-play/use areas with plants other than bermudagrass will interfere with the ability of these areas to serve as reservoirs of bermudagrass mite populations to infest high-value turf on golf courses.

Current bermudagrass mite management strategies that rely solely on miticides are ineffective, necessitating the identification of additional management tactics. Additionally, miticides proven effective at managing bermudagrass mite infestation are only available on golf courses and professional and collegiate sports fields (Boeri et al. 2018). Therefore, turfgrass managers of other sports fields, sod farms, and lawns must rely on cultural control strategies to manage bermudagrass mite infestation. Ensure adequate irrigation (>60% ET rate) to mite-infested turfgrass and lower nitrogen inputs to a level that maintains turfgrass quality but does not exacerbate mite infestation (≤24.5 kg N/ha every 4 wk). Similar strategies may also improve management programs on golf courses, though miticide application will likely also be necessary to reduce mite infestation to the low levels tolerable on golf courses.
